# Mortality prediction using a novel combination of biomarkers in the first day of sepsis in intensive care units

**DOI:** 10.1038/s41598-020-79843-5

**Published:** 2021-01-14

**Authors:** Junkun Liu, Chengwen Bai, Binbin Li, Aijun Shan, Fei Shi, Can Yao, Yu Zhang, Jin Wang, Weibu Chen, Manying Xie, Dehui Deng

**Affiliations:** 1 Emergency Department, Shenzhen People’s Hospital (The First Affiliated Hospital of Southern University of Science and Technology, The Second Clinical Medical College of Jinan University), Shenzhen, Guangdong China; 2 Dermatology Department, Xiangya Medical College, Central South University, Changsha, Hunan China; 3 Laboratory Department, Shenzhen People’s Hospital (The First Affiliated Hospital of Southern University of Science and Technology, The Second Clinical Medical College of Jinan University), Shenzhen, Guangdong China

**Keywords:** Prognostic markers, Diseases

## Abstract

Early identification of infection severity and organ dysfunction is crucial in improving outcomes of patients with sepsis. We aimed to develop a new combination of blood-based biomarkers that can early predict 28-day mortality in patients with sepsis or septic shock. We enrolled 66 patients with sepsis or septic shock and compared 14 blood-based biomarkers in the first 24 h after ICU admission. The serum levels of interleukin-6 (IL-6) (median 217.6 vs. 4809.0 pg/ml, *P* = 0.001), lactate (median 2.4 vs. 6.3 mmol/L, *P* = 0.014), N-terminal prohormone of brain natriuretic peptide (NT-proBNP) (median 1596.5 vs. 32,905.3 ng/ml, *P* < 0.001), prothrombin time (PT) (median 15.6 vs. 20.1 s, *P* = 0.030), activated partial thrombin time (APTT) (median 45.1 vs. 59.0 s, *P* = 0.026), and international normalized ratio (INR) (median 1.3 vs. 1.8, *P* < 0.001) were significantly lower in the survivor group. IL-6, NT-proBNP, and INR provided the best individual performance in predicting 28-day mortality of patients with sepsis or septic shock. Furthermore, the combination of these three biomarkers achieved better predictive performance (AUC 0.890, *P* < 0.001) than conventional scoring systems. In summary, the combination of IL-6, NT-proBNP, and INR may serve as a potential predictor of 28-day mortality in critically ill patients with sepsis or septic shock.

## Introduction

Sepsis is defined as life-threatening organ dysfunction caused by a dysregulated host response to infection^1^. It is a pathological syndrome caused by definite or suspected infection, and carries more than the mortality rates of 30%^2^. So far, it remains one of the leading causes of death worldwide in people of all ages, and the pathophysiological processes of this disease are well accepted. It involves not only the complex effects of systemic inflammation and immune dysfunction, but also acute failure of multiple organ systems in the body^3^. The immune response to infective agents may trigger a cascade of cytokines like interleukin-6 (IL-6), causing cells impairment, organs failure, and ultimately result in the coagulopathy, which is significantly correlated to the prognosis of sepsis patients^4^. As the condition deteriorates, hypotension and tachycardia can occur due to cardiovascular disorders, and serum levels of N-terminal prohormone of brain natriuretic peptide (NT-proBNP) are seen to be elevated in patients with myocardial depression, and often associated with a poor prognosis^5,6^. Moreover, it has been shown that serum levels of lactate and procalcitonin (PCT), Sequential Organ Failure Assessment (SOFA), and Acute Physiology and Chronic Health Evaluation II (APACHE II) scores are predictive of fatal outcomes in patients with critical illness^7^. Currently, there is still a lot of ambiguity surrounding the risk stratification and prognostic evaluation of comprehensive indicators for sepsis. Therefore, it may provide great value for patients with sepsis by establishing an accurate multi-index joint detection system.

The severity of infection and reversibility of organ dysfunction strongly affect the outcomes of sepsis patients. Although some studies have evaluated the prognostic value of some biomarkers such as lactate^8,9^, most of them focused only on inflammation or one organ failure, and the comprehensive assessment of biomarkers involving inflammatory states and different organ functions was rarely performed. In this study, we demonstrate the prognostic significance of several serological indicators such as IL-6, PCT, NT-proBNP, lactate, hepatorenal function, and coagulation function, to provide a clinical screening tool to identify and initiate early management of patients with sepsis.

## Materials and methods

This prospective, observational study was conducted on patients directly admitted to the Emergency intensive care unit (ICU) via Emergency Room, Shenzhen People’s Hospital, the first affiliated hospital of Southern University of Science and Technology, the second clinical medical college of Jinan University, from April 2017 to December 2018. In addition, all patients and/or family members signed informed consent and in accordance with the Ethical Guidelines of the Helsinki Declaration. This study was carried out with the approval of the Ethics Committee of Shenzhen People’s Hospital, the Second Clinical Medical College of Jinan University (NO. LL-KT-201705001).

### Study population

**Figure 1:**
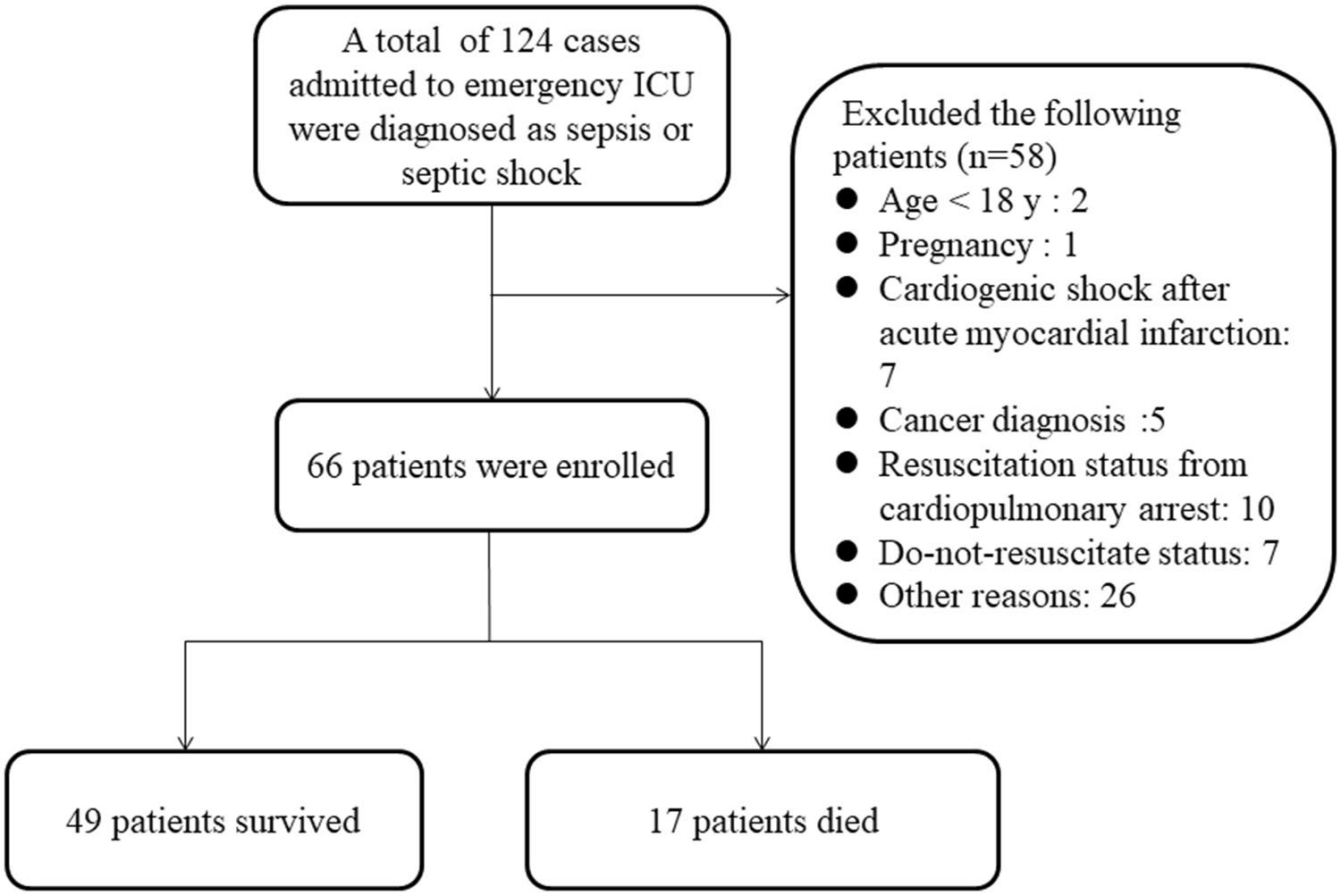
Screening and enrollment of the study.

Adult patients (≥ 18 years) admitted for sepsis or septic shock, according to Sepsis-3 criteria, were enrolled within 24 h of admission to the emergency ICU (Fig. 1). Following cases were excluded from the study: (1) under 18 years of age, (2) pregnancy, (3) shock of other origin, (4) acute coronary syndrome, (5) cancer, (6) resuscitation status from cardiopulmonary arrest, (7) trauma, (8) a requirement for immediate surgical intervention, (9) antibacterial therapy in the 5 days prior to enrollment, (10) a transfer to another hospital within 24 h after ICU admission, (11) having a slow progression of the disease over 7 days, and (12) those on do-not-resuscitate status. All subjects were given timely initial interventions, including appropriate antibiotics, hemodynamic resuscitation, and organ support therapy using protective mechanical ventilation or hemodialysis according to the Surviving Sepsis Campaign (SSC) 2016 guideline recommendations.

### Biomarker measurements

Blood samples were collected during first 24 h at ICU admission, and stored frozen until biomarker assays were performed (per manufacturer’s instructions) in the Laboratory Department of Shenzhen People's Hospital. The serum levels of 14 biomarkers were assayed: immunoturbidimetry method (Roche) for high-sensitivity C-reactive protein (hs-CRP), enzyme-linked fluorescent assay (VIDAS) for PCT, troponin I (Tn-I) and D-dimer, electro-chemiluminescence immunoassay (Roche) for IL-6 and NT-proBNP, colorimetric assay (Roche) for serum lactate, enzymatic colorimetric method (Roche) for serum creatinine (Cr), colorimetric diazo method (Roche) for total bilirubin, impedance and single light-scatter method for platelet (PLT) counting, coagulation tests (Sysmex) for prothrombin time (PT), activated partial thrombin time (APTT) and fibrinogen (Fib), and international normalized ratio (INR) was calculated from the following formula: INR = patient PT/mean normal PT) ^International Sensitivity Index (ISI). If the levels of biomarkers were measured multiple times in the first 24 h, the average level of each biomarker was used in our study.

### Data collection

We collected the following data: baseline demographic information, comorbidities, use of vasopressors, use of mechanical ventilator (MV), use of dialysis, length of hospital and ICU stay; mortality in 28 days; clinical indicators including mean arterial blood pressure (MAP), Glasgow Coma Scale (GCS)^10^, SOFA^11^, and APACHEII scores^12^. These scoring systems were calculated based on the data obtained during the first 24 h after ICU admission. Furthermore, the baseline pulse-induced contour cardiac output (PICCO) parameters of cardiac preload including the intrathoracic blood volume (ITBV) and global end diastolic volume (GEDV) were also collected. The follow-up phone calls were conducted with all subjects discharged from the hospital to collect the information for assessing 28-day mortality.

### Statistical evaluation

Statistical Package for Social Sciences (SPSS), version 19.0 (SPSS Inc, Chicago, IL) was used for statistical evaluations, and then the normal distribution of the data was confirmed by Kolmogorov–Smirnov test. Normally and non-normally distributed continuous variables were summarized as the mean (standard deviation, SD) and as the median (interquartile range, IQR), respectively. Data was analyzed by T test, post hoc LSD test or Mann–Whitney U test when applicable. The chi-squared test was for categorical variables. The univariate and multivariate logistic regression analyses were used to analyze the independent effects of various parameters on 28-day mortality. In order to identify the correlation between blood biomarkers and APACHEII or SOFA, variables were analysed using the Pearson correlation coefficients. The Receiver operating characteristic (ROC) curves were performed to predict the association of biomarkers with 28-day mortality in patients with sepsis^13^. A *P* value of less than 0.05 (*P* < 0.05) was considered as a statistically significant difference.

## Results

### Baseline characteristics and outcomes

A total of 66 patients with sepsis or septic shock were enrolled in this study after screened for eligibility. According to 28-day follow-up, 66 patients with sepsis were divided into survivor group (n = 49, 30 males and 19 females), and non-survivor group (n = 17, 9 males and 8 females). Among them, the average length of hospital and ICU stay were 11.4 ± 8.4 days and 5.5 ± 5.8 days respectively. The 28-day overall mortality was 25.8%. Inter-group comparisons revealed statistically significant differences in APACHE II (non-survivors vs. survivors: 36.5 ± 7.6 vs. 25.1 ± 8.8, *P* < 0.001), SOFA (non-survivors vs. survivors: 9.9 ± 4.6 vs. 6.2 ± 3.6, *P* = 0.001), and GCS scores (non-survivors vs. survivors: 7.1 ± 4.2 vs. 11.8 ± 4.4, *P* < 0.001). Furthermore, non-survivors had a greater risk of cardiovascular disease as a previous comorbidity (non-survivors vs. survivors: 47.1% vs. 18.4%, *P* = 0.020), and had a higher probability of mechanical ventilation (non-survivors vs. survivors: 64.7% vs. 20.4%, *P* = 0.001). While age, gender, MAP, etiology, and duration of hospital stay showed no statistical differences between the two groups. The comparisons between survivors and non-survivors are depicted in Table 1.

**Table 1:** Demographic characteristics at ICU admission.

Characteristics	Survivor group (n = 49)	Non-survivor group (n = 17)	Total (n = 66)	*P* value
Age, median (IQR)	69 (54–82)	77 (63–84)	73 (55.8–82)	0.099
Range	26–97	50–97	26–97	
Sex, male, n (%)	30 (61.2)	9 (52.9)	39 (59.1)	0.606
BMI, mean (SD)	24.5 (2.3)	23.8 (2.2)	24.4 (2.2)	0.766
MAP (mmHg), mean (SD)	82.5 (15.2)	86.9 (16.3)	83.6 (15.5)	0.311
**Comorbidity**				
Diabetes mellitus, n (%)	20 (40.8)	5 (29.4)	25 (37.9)	0.404
Hypertension, n (%)	25 (51.02)	11 (64.70)	36 (54.5)	0.329
Cardiovascular disease, n (%)	9 (18.4)	8 (47.1)	17 (25.8)	0.020*
Chronic renal failure, n (%)	17 (34.7)	7 (41.2)	24 (36.4)	0.632
Chronic lung disease, n (%)	6 (12.2)	3 (17.6)	9 (13.6)	1.0
Use of vasopressors, n (%)	6 (12.2)	3 (17.6)	9 (13.6)	0.881
Use of mechanical ventilation, n (%)	10 (20.4)	11 (64.7)	21 (31.8)	0.001*
Use of dialysis, n (%)	4 (8.2)	4 (23.5)	8 (12.1)	0.214
**Sepsis etiology**				
Pneumonia, n (%)	32 (65.3)	14 (82.4)	46 (69.7)	0.312
Urinary tract infection, n (%)	20 (40.8)	3 (17.6)	23 (34.8)	1.152
Intra-abdominal infection, n (%)	9 (18.4)	3 (17.6)	12 (18.2)	1.0
Soft tissue infection, n (%)	3 (6.1)	0 (0)	3 (4.5)	0.563
**Clinical scoring**				
SOFA, mean (SD)	6.2 (3.6)	9.9 (4.6)	7.2 (4.2)	0.001*
APACHE II score, mean (SD)	25.1 (8.8)	36.5 (7.6)	28.1 (9.9)	0.000*
GCS, mean (SD)	11.8 (4.4)	7.1 (4.2)	10.6 (4.8)	0.000*
**PICCO parameters**				
GEDI (ml/m^2^), mean (SD)	764.7 (234.0)	779.5 (75.0)	770.6 (179.9)	0.890
ITBV (ml/m^2^), mean (SD)	914.5 (282.5)	915.0 (97.1)	914.7 (217.9)	0.997
Hospital stay (day), mean (SD)	12.1 (7.1)	9.5 (11.6)	11.4 (8.4)	0.392

*IQR* range interquartile, *BMI* body mass index, *MAP* mean arterial pressure, *SOFA* sequential organ failure assessment, *GCS* Glasgow coma scale, *APACHE II* acute physiology and chronic health score II, *PICCO* Pulse-induced contour cardiac output, *GEDI* Global end-diastolic blood volume, *ITBV* Intrathoracic blood volume, *ICU* intensive care unit.

*Indicates a significant value, *P* < 0.05.

### Comparison of blood-based biomarkers between survivor and non-survivor groups

The serum levels of IL-6 [survivors vs. non-survivors: 217.6 (103.3–962.4) vs. 4809.0 (247.2–5000.0) pg/ml, *P* = 0.001], lactate [survivors vs. non-survivors: 2.4 (1.8–3.2) vs. 6.3 (2.5–14.3) mmol/L, *P* = 0.014], and NT-proBNP [survivors vs. non-survivors: 1596.5 (1708.6–10,635.4) vs. 32,905.3 (17,942.5–35,000.0) ng/ml, *P* < 0.001] were significantly lower in survivor group when compared to the non-survivor group, as shown in Table 2. There were no differences in serum levels of hs-CRP, PCT, Tn-I, Cr, and total bilirubin (*P* = 0.849, 0.961, 0.953, 0.215, 0.505, respectively) between the two groups. Since sepsis frequently is accompanied by coagulation abnormalities, we also compared coagulation function between non-survivors and survivors. We found that non-survivors had higher levels of PT [non-survivors vs. survivors: 20.1 (16.9–25.2) vs. 15.6 (13.2–17.4) s, *P* = 0.030], APTT [non-survivors vs. survivors: 59.0 (46.8–90.5) vs. 45.1 (38.5–55.3) s, *P* = 0.026], and INR [non-survivors vs. survivors: 1.8(1.5–3.8) vs. 1.3(1.1–1.5), *P* < 0.001] as compared to survivors. There were no significant differences with respect to other coagulation function indicators, including PLT, Fib, and d-dimer levels (*P* = 0.919, 0.882, 0.717, respectively).

**Table 2:** Biomarkers in blood of the study groups.

Blood biomarkers	Survivor group (n = 49)	Nor-survivor group (n = 17)	Total (n = 66)	*P* value
**Inflammation**				
hs-CRP (mg/L), median (IQR)	188.3 (102.9–295.7)	175.7 (136.5–300.2)	187.9 (129.8–296.8)	0.849
PCT (ng/ml), median (IQR)	28.0 (6.1–95.3)	29.0 (10.8–102.4)	28.5 (6.3–97.1)	0.961
IL-6 (pg/ml), median (IQR)	217.6 (103.3–962.4)	4809.0 (247.2–5000.0)	313.7 (121.3–2565.3)	0.001*
**Circulation**				
Lactate (mmol/l), median (IQR)	2.4 (1.8–3.2)	6.3 (2.5–14.3)	2.5 (1.9–4.1)	0.014*
NT-proBNP (ng/ml), median (IQR)	1596.5 (1708.6–10,635.4)	32,905.3 (17,942.5–35,000.0)	3720.1 (880.9–23,665.7)	< 0.001*
Tn-I (ng/ml), median (IQR)	0.0 (0.01–0.12)	0.2 (0.01–0.98)	0.01 (0.01–0.31)	0.953
**Renal function**				
Cr (μmol/l), median (IQR)	125.0 (92.0–247.0)	252.0 (239.0–463.0)	170.0 (103.0–281.0)	0.215
**Liver function**				
Total bilirubin (μmol/l), median (IQR)	13.8 (5.0–70.9)	14.8 (8.3–110.9)	14.0 (6.0–88.9)	0.505
**Coagulation function**				
PLT (× 10^9^/l), mean (SD)	170.6 (109.5)	167.4 (110.5)	169.7 (108.0)	0.919
PT (s), median (IQR)	15.6 (13.2–17.4)	20.1 (16.9–25.2)	16.2 (14.0–19.5)	0.030*
APTT (s), median (IQR)	45.1 (38.5–55.3)	59.0 (46.8–90.5)	49.0 (42.2–58.0)	0.026*
Fib (g/l), median (IQR)	5.1 (4.2–7.7)	5.7 (2.2–6.2)	5.1 (4.1–7.3)	0.882
INR, median (IQR)	1.3 (1.1–1.5)	1.8 (1.5–3.8)	1.4 (1.2–1.8)	< 0.001*
d-Dimer (μg/l), median (IQR)	3948.5 (2093.5–7848.0)	8889.4 (4278.9–10,000.0)	4765.9 (2792.7–8716.9)	0.717

*IQR* range interquartile, *hs-CRP* high-sensitivity C-reactive protein, *PCT* procalcitonin, *IL-6* Interleukin 6, *NT-proBNP* N-terminal prohormone of brain natriuretic peptide, *Tn-I* Troponin I, *Cr* creatinine, *PLT* Platelet, *PT* prothrombin time, *APTT* activated partial thrombin time, *Fib* fibrinogen, *INR* international normalized ratio.

*Indicates a significant value, *P* < 0.05.

### Logistic regression analysis of blood-based biomarkers associated with 28-day mortality

In this study, the variables of IL-6, lactate, NT-proBNP, PT, APTT, and INR for which *P* <  0.05 between non-survivors and survivors were included. In the logistic regression analysis, the levels of IL-6 [odds ratio (OR) 1.001; 95% confidence interval (CI) 1.000–1.001; *P* = 0.010], NT-proBNP (OR 1.000; 95% CI 1.000–1.000; *P* = 0.013), and INR (OR 1.917; 95% CI 0.949–3.871; *P* = 0.046) within the first 24 h of admission were independently associated with 28-day mortality of sepsis (Table 3), and were valuable for predicting 28-day mortality. However, other variables including lactate, PT, and APTT were not significant predictors of 28-day mortality.

**Table 3:** The logistic regression analysis predicting 28-day mortality in patients with sepsis or septic shock.

Variables	Univariable analysis			Multivariable analysis		
	OR	95% CI	*P* value	OR	95% CI	*P* value
INR	2.825	1.326–6.020	0.007*	1.917	0.949–3.871	0.046*
NT-proBNP	1.000	1.000–1.000	0.000*	1.000	1.000–1.000	0.013*
IL-6	1.001	1.000–1.001	0.000*	1.001	1.000–1.001	0.010*
Lactate	1.327	1.103–1.597	0.003*	–	–	–
PT	1.135	1.030–1.251	0.011	–	–	–
APTT	1.037	1.006–1.070	0.019	–	–	–
CRP	1.000	0.995–1.005	0.981	–	–	–
PCT	0.999	0.990–1.009	0.906	–	–	–
Tn-I	0.995	0.833–1.187	0.952	–	–	–
Cr	1.002	0.999–1.004	0.178	–	–	–
PLT	0.896	0.995–1.009	0.978			
Fib	0.898	0.712–1.133	0.363	–	–	–
d-Dimer	1.000	1.000–1.000	0.054	–	–	–

*OR* odds ratio, *CI* confidence interval, *INR* international normalized ratio, *NT-proBNP* N-terminal prohormone of brain natriuretic peptide, *IL-6* Interleukin 6, *PT* prothrombin time, *APTT* activated partial thrombin time, *hs-CRP* high-sensitivity C-reactive protein, *PCT* procalcitonin, *Tn-I* Troponin I, *Cr* creatinine, *PLT* Platelet, *Fib* fibrinogen.

*Indicates a significant value, *P* < 0.05.

### ROC curve analysis of blood-based biomarkers for predicting 28-day mortality

**Figure 2:**
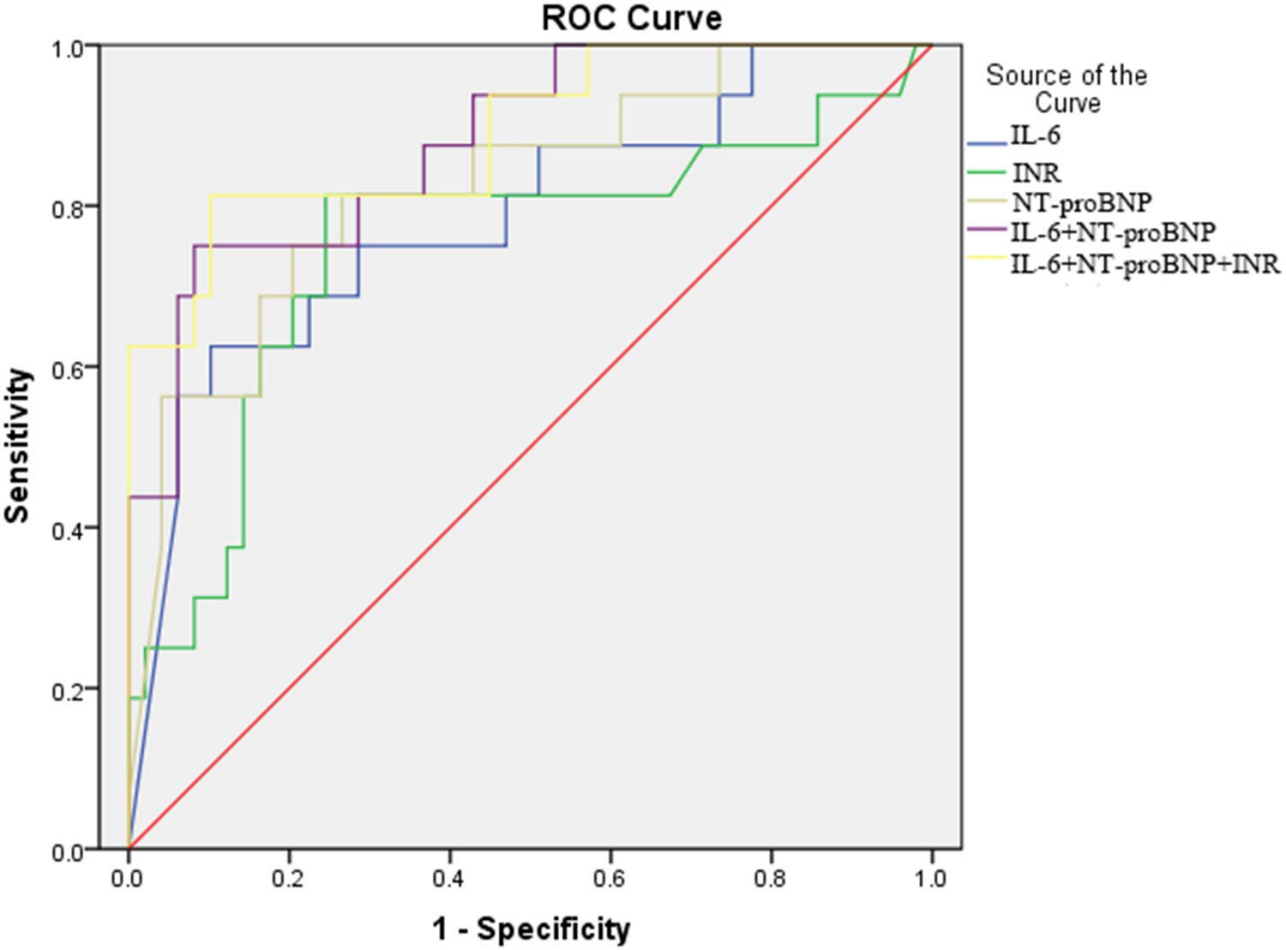
The receiver operating characteristic (ROC) curves of the selected biomarkers for predicting 28-day mortality of critically ill patients with sepsis or septic shock. The ROC curves for single biomarker and their combinations had the following areas: interleukin-6 (IL-6), 0.785; international normalized ratio (INR), 0.748; N-terminal pro-brain natriuretic peptide (NT-proBNP), 0.825; combination of IL-6 and NT-proBNP, 0.879; and combination of IL-6, INR and NT-proBNP, 0.890.

The ability of selected biomarkers, SOFA, and APACHE II scores to predict the 28-day mortality in patients with sepsis according to ROC curve analysis was shown in Table 4 and Fig. 2. The optimal cutoff value for IL-6 was 2580.5 pg/ml, with sensitivity of 63%, and specificity of 90% (AUC 0.785, 95% CI 0.647–0.923, *P* = 0.001); 3.75 mmol/l for lactate with 63% sensitivity and 84% specificity (AUC 0.776, 95% CI 0.633–0.918, *P* = 0.001); 9086.59 ng/ml for NT-proBNP with 70% sensitivity and 80% specificity (AUC 0.825, 95% CI 0.704–0.947, *P* < 0.001); 17.55 s for PT with 71% sensitivity and 78% specificity (AUC 0.700, 95% CI 0.529–0.870, *P* = 0.017); 44.50 s for APTT with 82% sensitivity and 47% specificity (AUC 0.698, 95% CI 0.552–0.844, *P* = 0.016); and 1.465 for INR with 81% sensitivity and 76% specificity (AUC 0.748, 95% CI 0.590–0.906, *P* = 0.003). Moreover, we also performed the combination tests to gain a better performance, and revealed that the combination of IL-6, NT-proBNP and INR (AUC 0.890, 95% CI 0.791–0.989, *P* < 0.001) had superiority in predicting 28-day mortality than that of SOFA (AUC 0.732, 95% CI 0.586–0.878, *P* = 0.005) and APACHE II scores (AUC 0.850, 95% CI 0.783–0.942, *P* < 0.001).

**Table 4:** Prognostic value of clinical parameters.

Blood biomarkers	AUC	95% CI	Cut-off	Sensitivity	Specificity	*P*-value
INR	0.748	0.590–0.906	1.465	0.81	0.76	0.003*
NT-proBNP (ng/ml)	0.825	0.704–0.947	9086.59	0.70	0.80	0.000*
IL-6 (pg/ml)	0.785	0.647–0.923	2580.5	0.63	0.90	0.001*
Lactate (mmol/l)	0.776	0.633–0.918	3.75	0.63	0.84	0.001*
PT (s)	0.700	0.529–0.870	17.55	0.71	0.78	0.017*
APTT (s)	0.698	0.552–0.844	44.50	0.82	0.47	0.016*
SOFA scores	0.732	0.586–0.878	7.5	0.71	0.76	0.005*
APACHE II scores	0.850	0.758–0.942	27.5	0.94	0.69	0.000*
IL-6 + NT-proBNP	0.879	0.783–0.974	–	0.75	0.92	0.000*
IL-6 + INR + NT-proBNP	0.890	0.791–0.989	–	0.81	0.90	0.000*

*AUC* Area under the curve, *CI* confidence interval, *IL-6* Interleukin 6, *INR* international normalized ratio, *NT-proBNP* N-terminal prohormone of brain natriuretic peptide, *PT* prothrombin time, *APTT* activated partial thrombin time, *SOFA* sequential organ failure assessment, *APACHE II* acute physiology and chronic health score II.

*Indicates a significant value, *P* < 0.05.

### Correlation analysis of biomarkers in the study groups

**Figure 3:**
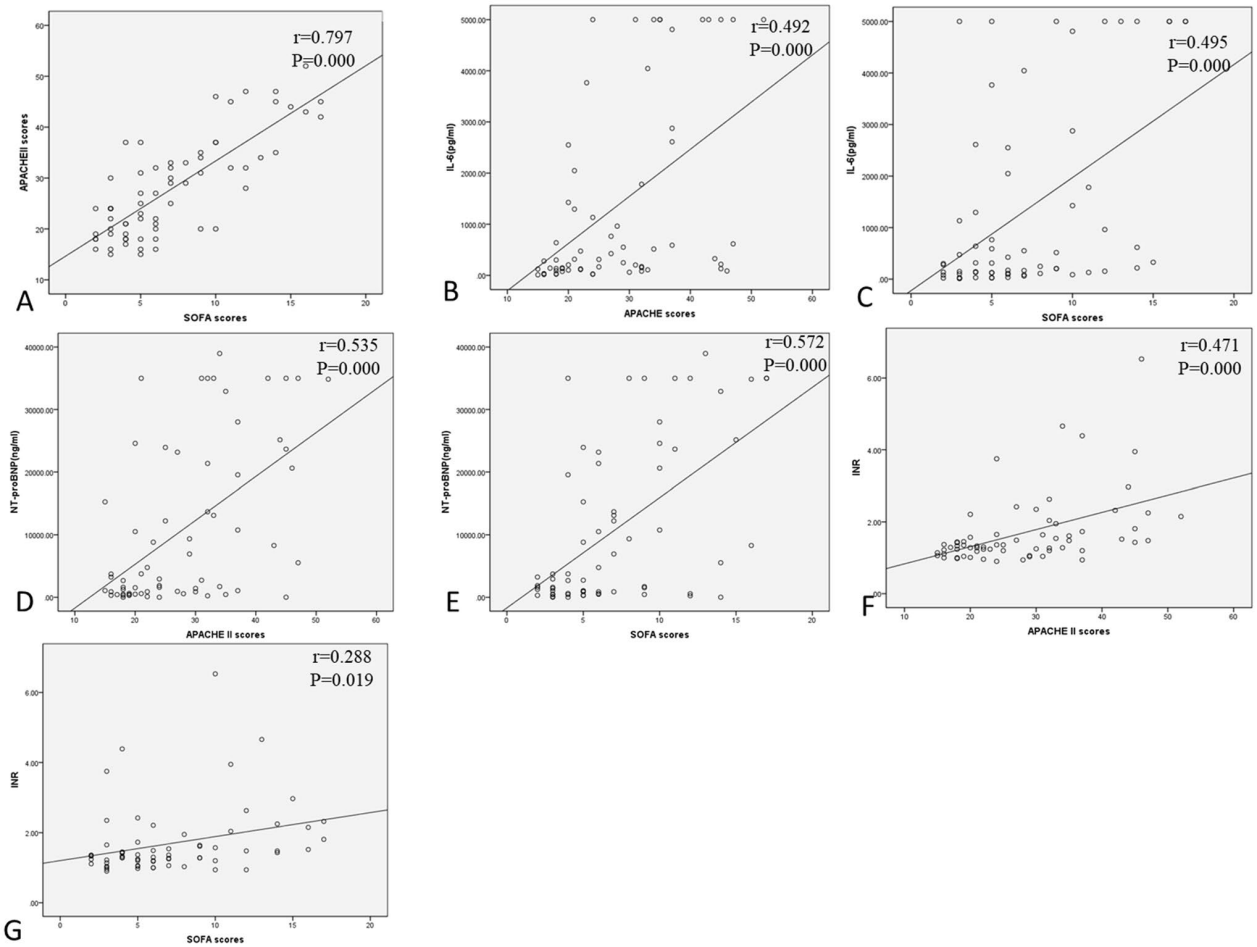
Correlation analysis of biomarkers in all subjects. (**A**) Correlation between APACHE II and SOFA, (**B**) Correlation between IL-6 and APACHE II, (**C**) Correlation between IL-6 and SOFA, (**D**) Correlation between NT-proBNP and APACHE II, (**E**) Correlation between NT-proBNP and SOFA, (**F**) Correlation between INR and APACHE II, and (**G**) Correlation between INR and SOFA.

The correlation coefficient analysis revealed a strong positive relationship between APACHEII and SOFA scores (r = 0.797, *P* < 0.001). Furthermore, the levels of IL-6, NT-proBNP, INR, lactate and PT in all patients was positively associated with APACHEII (r = 0.492, 0.535, 0.471, 0.371, 0.471, respectively; *P* all < 0.05) and SOFA scores (r = 0.495, 0.572, 0.288, 0.347, 0.355, respectively; *P* all < 0.05). The APTT levels were positively associated with APACHEII scores (r = 0.298, *P* = 0.015). However, we didn't find an association between APTT levels and SOFA scores (*P* = 0.222). More details were shown in Table 5 and Fig. 3.

**Table 5:** Correlations between blood biomarkers and APACHE II or SOFA scores in all subjects.

Blood biomarkers	APACHE II		SOFA	
	r value	*P* value	r value	*P* value
IL-6	0.492	0.000*	0.495	0.000*
NT-proBNP	0.535	0.000*	0.572	0.000*
INR	0.471	0.000*	0.288	0.019*
Lactate	0.371	0.002*	0.347	0.004*
PT	0.471	0.000*	0.355	0.003*
APTT	0.298	0.015*	0.152	0.222

*Indicates a significant value, *P* < 0.05.

*IL-6* Interleukin 6, *INR* international normalized ratio, *NT-proBNP* N-terminal prohormone of brain natriuretic peptide, *PT* prothrombin time, *APTT* activated partial thrombin time, *APACHE II* acute physiology and chronic health score II, *SOFA* sequential organ failure assessment.

## Discussion

We evaluated the inflammatory and organ-specific biomarkers to predict 28-day mortality for critically ill patients with sepsis, and found that a novel combination of INR, NT-proBNP and IL-6 within first 24 h of ICU admission may rapidly predict mortality risk in patients with sepsis or septic shock. This combination of three markers is solely based on cardiac dysfunction, coagulation disorders, and inflammatory responses, which are readily available during the patient's course of ICU stay. Therefore, it may help improve risk stratification, early assessment and intervention of sepsis patients in ICU. Our findings also suggest that the optimal combination of INR, NT-proBNP and IL-6 has a significantly higher AUC value than that of conventional scoring systems such as SOFA and APACHEII scores, and provides need to confirm its validity for screening sepsis-related mortality.

Sepsis is also associated with coagulopathy ranging from hypercoagulability to acute disseminated intravascular coagulation (DIC), which may be likely play a key role in multiple organ dysfunction syndrome (MODS)^14^. In particular, hemostasis-related parameter has been shown to be a predictor of poor outcomes in ICU patients. Thrombocytopenia occurs in up to 40% of patients on admission, and an INR ≥ 1.5 is present in approximately two-thirds of patients. We found that the INR value in survivors was significantly lower than in non-survivors, and strongly associated with greater risk of serious illness. Furthermore, multivariate analysis demonstrated that INR level was an independent factor for critically ill patients with sepsis, and high INR (> 1.47) was significantly associated with increased mortality risk. In fact, because coagulopathy (INR > 1.5) is defined as one of the critical characteristics in patients with sepsis according to sepsis 3.0 criteria, this definition would partly support our results^15^. However, some authors still questioned the use of INR as a management tool because INR value can be falsely increased, and not necessarily associated with risk of bleeding events^16^. Our study also showed that the INR value of 1.47 has a sensitivity of 81%, specificity of 76% and AUC of 0.748, which may limit its use alone at accurate assessment on the outcomes of sepsis patients.

Since NT-proBNP is mainly secreted by cardiomyocytes in response to increased ventricular volume or pressure load, the level of this biomarker is known to be highly sensitive to detect acute heart failure, and correlated with the severity of heart failure^17,18^. In addition, low-grade systematic inflammation and insulin resistance are other possible causes of elevated level of NT-proBNP in patients with sepsis^19^. Previous studies revealed the levels of NT-proBNP in sepsis patients without heart failure were associated with in-hospital mortality^20^. Therefore, it has been accepted that the increased levels of NT-proBNP in septic shock patients are mainly due to sepsis rather than cardiac insufficiency. In agreement with previous studies, we found that on the first day of ICU admission, the levels of NT-proBNP in non-survivor group were significantly higher than in survivor group, and the higher NT-proBNP levels were associated with poorer health status, including higher SOFA scores and higher APACHE II scores. Moreover, the volume challenge during resuscitation process might be a possible reason for functional heart failure, and had some influence on the increased levels of NT-proBNP. In this study, we also found that NT-proBNP also presented as an independent risk factor for sepsis mortality. At a cut-off value of 9086.59 ng/ml, NT-proBNP had a sensitivity of 70% and a specificity of 80% with an AUC of 0.825. These findings suggested NT-proBNP may be a potential predictor of sepsis mortality, but needs to increase its sensitivity and specificity by combining with other parameters.

Elevated levels of serum IL-6 have been found in both neonatal and adult sepsis^21,22^. Previous studies have shown that IL-6 may be induced by infections and tissue injuries, and associated with a high risk of death in septic shock^23^. However, IL-6 is a multifunctional cytokine that has pro- and anti-inflammatory properties, and can be affected by different conditions such as exercise, aging, certain cancers, frailty, and chronic inflammatory response^24^. These factors should be comprehensively considered for a better understanding of sepsis when IL-6 levels are elevated. In this study, we observed that the IL-6 levels measured on day 1 were significantly increased in non-survivors when compared with survivors, and positively correlated with SOFA and APACHEII scores. A cut-off value for IL-6 on 2580.5 pg/ml was found to have a moderate sensitivity of 63% and a high specificity of 90% (AUC 0.785) for predicting mortality. The results suggested IL-6 may be used as a prognostic biomarker for 28-day mortality in patients with sepsis, while the predictive accuracy of IL-6 is lower than that of NT-proBNP.

Various studies have demonstrated that the APACHE II and SOFA scoring systems are good tools for predicting mortality in ICU patients^25,26^. Unlike APACHE II system, the SOFA was not initially designed to predict hospital mortality, while it has been broadly used in a range of applications such as organ dysfunction assessment, predicting hospital survival, and identifying non-ICU patients at risk of sepsis. Furthermore, the specificities of APACHE II and SOFA scores were found to be somewhat low in identifying the outcomes in different subgroups of ICU patients^27^. In the present study, we also revealed that in predicting 28-day mortality, APACHE II score of > 27.5, and SOFA score > 7.5 performed at sensitivities of 94%, and 71%, and specificities of 69% and 76%, respectively. Even though APACHE II score performed well in mortality risk stratification in the ICU patients with sepsis, it tended to a higher sensitivity and lower specificity, leading to an overestimation of mortality risk (and consequently, a higher burden on hospital costs). Moreover, the combination of three biomarkers, INR, NT-proBNP and IL-6 yielded an ROC value of 0.890, which provided higher diagnostic accuracy (sensitivity of 81% and specificity of 90%) than any single biomarker, APACHE II and SOFA scores. This result showed that the three-marker combination may have the best performance in predicting sepsis-related mortality. Therefore, instead of just screening APACHE II and SOFA scores during the initial 24 h in the ICU, we prefer to monitor the three-marker combination to predict the short outcome of sepsis patients. Once the patient meets the cut-off levels of the combination, we should closely observe the changes of multiple physiological systems, and frequently adjust medical therapies accordingly to reduce the risk of death. Furthermore, these three markers together may help to early stage the disease, predict prognosis and response of intervention in critically ill patients with sepsis.

Our study has several limitations. First, it was a single-center study, and the small size of study population is the major limitation. Therefore, larger observations of multiple centers should be carried out to verify the results. Second, we did not include other clinical parameters such as arterial blood gas analysis and white blood cell count, because these variables have not shown a sufficient predictive power for the outcomes of sepsis patients. Future studies need to elucidate the potential application of other clinical biomarkers for prognosis in sepsis, including but not limited to the parameters associated with dysfunction of major organs.

Despite the above limitations, our findings indicate the combination of INR, NT-proBNP and IL-6 may represent a valuable tool for predicting mortality risk in critically ill patients with sepsis or septic shock. Furthermore, this simple combination shows a higher prognostic accuracy for 28-day mortality than the SOFA or APACHE II scores, and could have a potential application in guiding early management of sepsis patients in ICU. Future studies are necessary to explore whether the combination can improve patient managements and outcomes in ICU.

## Data Availability

Some or all data, or code generated or used during the study are available from the corresponding author by request.
